# Supplementary material for: Differences in the performance of resuscitation according to the resuscitation guideline terminology during infant cardiopulmonary resuscitation: “Approximately 4 cm” versus “at least one-third the anterior-posterior diameter of the chest”
Source: PLoS One. 2020 Mar 24;15(3):e0230687. doi: 10.1371/journal.pone.0230687 (PMC7092967; doi:10.1371/journal.pone.0230687)
Supplement: Attachment — Submitted filename: Response to the reviewers comments.docx [file pone.0230687.s003.docx]

4 March 2020

Kevin Ching, MD

Academic Editor
*PLOS ONE*

Dear Editor,

I wish to re-submit the manuscript titled “Differences in the performance of resuscitation according to the resuscitation guideline terminology during infant cardiopulmonary resuscitation: “approximately 4 cm” versus “at least one-third the anterior-posterior diameter of the chest”.” The manuscript ID is PONE-D-19-34142_R1.

We thank you and the reviewers for your thoughtful suggestions and insights. The manuscript has benefited from these insightful suggestions. I look forward to working with you and the reviewers to move this manuscript closer to publication in *PLOS ONE*. The manuscript has been rechecked and the necessary changes have been made in accordance with the reviewers’ suggestions. Thank you for your consideration. I look forward to hearing from you.

Sincerely,

Je Hyeok Oh, MD, PhD

Department of Emergency Medicine,

Chung-Ang University College of Medicine,

84 Heukseok-ro, Dongjak-gu, Seoul 06974, Republic of Korea

Tel. no.: +82 2 6299 1820

Fax no.: +82 2 6264 1119

Email address: [jehyeokoh@cau.ac.kr](mailto:jehyeokoh@cau.ac.kr)

**Our response to the comments of the Reviewer #1:**

1. Thank you for your edits. The revised manuscript has addressed our concerns. One minor additional question: What specialty of interns and residents were enrolled? We only know that they were certified in BLS. It is possible that the specialty of residents might impact on their CCD. For instance, a pediatric resident (who has more confidence with children) might be more aggressive than an internal medicine resident, leading to deeper compressions by the pediatric residents. If the distribution of different specialities between Group A and B are unequal, this could bias the results.

Answer) Thank you for your valuable comments. We have revised the manuscript according to the reviewer’s recommendation. In detail, there were no specialities in the intern physician. In South Korea, intern physicians rotate all clinical fields (specialities) for one year before starting the course of resident physician. Therefore, we have compared whether there was a difference in the specialities of the resident physician according to the groups. As a result, there were no differences in the distribution of specialities between Group A and B. We have added the results in the table 1.

Table 1. Characteristics of the study participant according to the study group

| Variables | Group A (n = 20) | Group B (n = 20) | *P*-value |
| --- | --- | --- | --- |
| Male sex (%) | 15 (75.0) | 15 (75.0) | 1.000 |
| Age (years) | 27.5 (26.0, 29.0) | 26.5 (25.0, 29.5) | 0.529 |
| Position |  |  | 1.000 |
| Resident physician | 11 (55.0) | 11 (55.0) |  |
| Intern physician | 9 (45.0) | 9 (45.0) |  |
| Specialities of the resident physician |  |  | 0.830 |
| Emergency Medicine | 3 (27.3) | 4 (36.4) |  |
| Internal Medicine | 4 (36.4) | 3 (27.3) |  |
| Pediatrics | 2 (18.2) | 0 (0.0) |  |
| General Surgery | 0 (0.0) | 2 (18.2) |  |
| Orthopedics | 0 (0.0) | 1 (9.1) |  |
| Dermatology | 1 (9.1) | 1 (9.1) |  |
| Psychiatrics | 1 (9.1) | 0 (0.0) |  |
| Right handedness (%) | 18 (90.0) | 19 (95.0) | 1.000 |

Data are expressed as n (%) except for age, which is presented as median and interquartile range.

Sincerely yours,

Je Hyeok Oh, MD, PhD
